# Molecular characterization of influenza viruses collected from young children in Uberlandia, Brazil - from 2001 to 2010

**DOI:** 10.1186/s12879-015-0817-z

**Published:** 2015-02-18

**Authors:** Thelma Fátima de Mattos Silva Oliveira, Jonny Yokosawa, Fernando Couto Motta, Marilda Mendonça Siqueira, Hélio Lopes da Silveira, Divina Aparecida Oliveira Queiróz

**Affiliations:** Laboratório de Virologia, Instituto de Ciências Biomédicas, Universidade Federal de Uberlândia (UFU), Uberlândia, MG Brazil; Faculdade de Medicina – UFU, Uberlândia, MG Brazil; Laboratório de Vírus Respiratórios, Instituto Oswaldo Cruz, Fiocruz, RJ Brazil

**Keywords:** Influenza virus, Children, RT-PCR, Sequencing, Hemagglutinin, Neuraminidase

## Abstract

**Background:**

Influenza remains a major health problem due to the seasonal epidemics that occur every year caused by the emergence of new influenza virus strains. Hemagglutinin (HA) and neuraminidase (NA) glycoproteins are under selective pressure and subjected to frequent changes by antigenic drift. Therefore, our main objective was to investigate the influenza cases in Uberlândia city, Midwestern Brazil, in order to monitor the appearance of new viral strains, despite the availability of a prophylactic vaccine.

**Methods:**

Nasopharyngeal samples were collected from 605 children less than five years of age presenting with acute respiratory disease and tested by immunofluorescence assay (IFA) for detection of adenovirus, respiratory syncytial virus, parainfluenza virus types 1, 2, and 3 and influenza virus types A and B. A reverse transcription-PCR (RT-PCR) for influenza viruses A and B was carried out to amplify partial segments of the HA and NA genes. The nucleotide sequences were analyzed and compared with sequences of the virus strains of the vaccine available in the same year of sample collection.

**Results:**

Forty samples (6.6%) were tested positive for influenza virus by IFA and RT-PCR, with 39 samples containing virus of type A and one of type B. By RT-PCR, the type A viruses were further characterized in subtypes H3N2, H1N2 and H1N1 (41.0%, 17.9%, and 2.6%, respectively). Deduced amino acid sequence analysis of the partial hemagglutinin sequence compared to sequences from vaccine strains, revealed that all strains found in Uberlândia had variations in the antigenic sites. The sequences of the receptor binding sites were preserved, although substitutions with similar amino acids were observed in few cases. The neuraminidase sequences did not show significant changes. All the H3 isolates detected in the 2001-2003 period had drifted from vaccine strain, unlike the isolates of the 2004-2007 period.

**Conclusions:**

These results suggest that the seasonal influenza vaccine effectiveness could be reduced because of A H3N2 variants that circulated in 2001-2003 years. Thus, an early monitoring of variants circulating in the country or in a region may provide important information about the probable efficacy of the vaccine that will be administered in an influenza season.

**Electronic supplementary material:**

The online version of this article (doi:10.1186/s12879-015-0817-z) contains supplementary material, which is available to authorized users.

## Background

Influenza viruses are pathogens responsible for causing respiratory disease worldwide and represent a major threat to public health due to annual epidemics and the potential of pandemics. These agents are members of Orthomyxoviridae family and are classified into types A, B and C. Influenza viruses of type A can be further divided into subtypes based on the antigenic properties of the surface glycoproteins hemagglutinin (HA) and neuraminidase (NA) [1]. Currently, 18 HA and 11 NA variants are known [2] and they are used to subtype the influenza virus isolates, with subtypes H1N1 and H3N2 characterized by the sustained transmission in the human population [3]. HA and NA are under selective pressure and undergo frequent changes through antigenic drift, resulting in the appearance of new strains that evade the host immune system [4]. Distinct antigenic sites located in the H1 sequence (Sa, Sb, Ca and Cb) and five major variable sites within domain 1 of H3 (A through E) are targets of neutralizing antibodies and virus isolates carrying substitutions at these sites have been related with epidemics in humans in limited time periods [5,6]. Vaccination is the main strategy to reduce the public health burden caused by influenza. However, its effectiveness depends on how close the HA sequences of the virus strains used in the vaccine are to those circulating in the same influenza season [7]. Sequence analysis of the glycoprotein genes allows the identification of new strains and may also reveal geographical regions where the detected strains have circulated. Therefore, knowledge of the circulating strains and genetic analysis of HA and NA are invaluable to guide effective measures for influenza prevention and control. In Brazil, influenza virus sequences and strain information are currently available from only a few locations and surveillance efforts have been increased after the emergence of the pandemic influenza A/H1N1 virus in humans, in 2009 [8]. In Uberlândia, in the southeastern region of the country, however, information about circulating strains is not known. Thus, the purposes of this study were to detect the influenza viruses in young children presenting with acute respiratory disease, to characterize the strains from the identified cases and to compare the HA and NA sequences of these strains with the sequences of the virus variants used in the vaccines of the same flu season.

## Methods

### Specimens

Nasopharyngeal aspirates (NPA) were collected from children under five years old presenting with acute respiratory disease (ARD), within five days of onset of clinical symptoms, as described by Costa et al. [9]. The collection of clinical specimens was performed in public health service units in Uberlândia city, in the state of Minas Gerais, southeastern Brazil, from 2001 to 2010 and aliquots from the specimens were stored at −70°C. Initially, the specimens were screened by immunofluorescence assay (IFA) with the Respiratory Panel I Viral Screening and Identification Kit (Millipore/Chemicon International, Inc., Temecula, CA, USA), following the manufacturer’s instructions, to detect the presence of influenza viruses A and B, respiratory syncytial viruses A and B, parainfluenza virus types 1, 2 and 3 and adenovirus. RT-PCR for detection of influenza virus RNA was used with all samples that tested positive for influenza virus by IFA and, depending on the availability, with IFA-negative or -inconclusive samples. The study was approved by the Ethics Committee of Universidade Federal de Uberlândia (protocol number 326/08) and a written consent was obtained by one of the patient’s parents.

### RNA extraction and RT-PCR

Total RNA was extracted from 140 μL NPA by using Viral RNA mini kit (Qiagen, Hilden, Germany) according to the manufacturer’s recommendations.

For reverse transcription, the reaction was carried out in a total volume of 20 μL, which contained 5 μL total RNA, 0.5 mM each deoxynucleoside triphosphate, 1 μM primer for RT (Table 1) [10], 0.1 M DTT, 100 U SuperScript™ III Reverse Transcriptase (Life Technologies Corp, Grand Island, NY, USA), and buffer supplied by the enzyme manufacturer. The first three components were mixed, incubated at 95°C for 3 min and cooled on ice for 1 min. Then, the other components were added to the tube, the volume was completed with diethyl pyrocarbonate (DEPC)-treated water, the mixture was incubated at 50°C for 60 min, and the enzyme was inactivated at 95°C for 3 min. Four μL cDNA was submitted to first-round PCR, in a total reaction volume of 20 μL, which contained 1 mM MgCl_2_, 0.2 mM of each deoxyribonucleoside triphosphate, 1 or 0.25 μM each primer (for HA or NA sequence amplification, respectively) (each sequence was amplified separately), 1 U of Platinum *Taq* DNA polymerase (Life Technologies) and buffer supplied by the enzyme manufacturer. The reaction was carried out with the following conditions: 94°C for 1 min; 35 cycles of 94°C for 1 min, 50°C or 55°C (respectively, for HA or NA sequence amplification) for 1 min, and 72°C for 1.5 min. The first-round amplification product was diluted 1:100 in water and 0.8 μL was submitted to a second-round amplification, with similar conditions used in the first-round PCR, except for the following: primer concentrations were changed to 0.125 or 0.25 μM (HA or NA, respectively), MgCl_2_ to 1.5 mM, and annealing temperature to 52 or 57°C (HA or NA, respectively) for 1 min. PCR products were separated in a 1.0% agarose gel in tris-borate-EDTA buffer [11], stained with GelRed ^TM^ (Biotium, Hayward, CA, USA) and visualized under ultraviolet light.

**Table 1:** **Sequences of the oligonucleotides used for cDNA synthesis, PCR amplification and sequencing reactions**

**Primer**	**Sequence 5′-3′**	**Specificity**	**Reaction(s)**	**Nested-PCR amplicon size (bp)**
AH1A	CAG ATG CAG ACA CAA TAT GT	H1	RT, 1st-round PCR	
AH1FII	AAA CCG GCA ATG GCT CCA AA			
AH1B	ATA GGC TAC CAT GCG AAC AA		2nd-round PCR	
AH1EII	CTT AGT CCT GTA ACC ATC CT			944
AH3A	CAG ATT GAA GTG ACT AAT GC	H3	RT, 1st-round PCR	
AH3DII	GTT TCT CTG GTA CAT TCC GC			
AH3B	AGC AAA GCT TTC AGC AAC TG		2nd-round PCR	
AH3CII	GCT TCC ATT TGG AGT GAT GC			591
BHAA	GTG ACT GGT GTG ATA CCA CT	B	RT, 1st-round PCR	
BHADII	TGT TTT CAC CCA TAT TGG GC			
BHAB	CAT TTT GCA AAT CTC AAA GC		2nd-round PCR	
BHACII	TGG AGG CAA TCT GCT TCA CC			767
AN1/418 F	TTC TGA CCC AAG GTG CTC TA	N1	RT, 1st- and 2nd-round PCR	
AN1/EII	TAC TTG TCA ATG GTG AAC GG		1st-round PCR	
AN1/1219R	GAA ACT TCC GCT GTA CCC TG	N2	2nd-round PCR	821
AN2A/Multi	AAC ATT ACT GGA TTT GCA CC		RT, 1st-round PCR	
AN2DII/1082	CAA AGG CCC AGC CTT TCA CT		1st- and 2nd-round PCR	
AN2B	GGT GAC GAG AGA ACC TTA TG		2nd-round PCR	719
H3.938.R	**TGT CAG AGG TTT TCA CCG TC**G CTT CCA TTT GGA GTG ATG C	HA1	890-909	
**Others**				
H3.101.F	CAG CAC GGC AAC GCT G	NA	101-116	
3GSTRV	TGT CAG AGG TTT TCA CCG TC			

### Nucleotide sequencing and analysis

The amplified products were purified by using GenElute PCR Clean-Up Kit (Sigma-Aldrich, Inc., St. Louis, MO, USA) and sequencing was performed by using either DYEnamic™ ET Dye Terminator Kit and MegaBACE 1000 sequencer (GE Healthcare, Buckinghamshine, UK) or BigDye Terminator v.3.1 and ABI-Prism 3100 Genetic Analyzer (Applied Biosystems/Life Technologies, Foster City, CA, USA), following the manufacturer’s instructions. Sequences were edited by using SeqMan Pro (Lasergene version 10, DNASTAR, Inc. Madison, WI, USA) and search of similar sequences was carried out using BLAST (http://blast.ncbi.nlm.nih.gov/Blast.cgi). Sequence alignment was performed by using the Clustal W method in MegAlign (DNASTAR). The nucleotide sequences of the characterized isolates in this study were submitted to GenBank under accession numbers KF918346 to KF918392.

## Results

A total of 605 clinical samples were collected from patients with ARD between 2001 and 2010 and tested by IFA. Thirty seven (6.1%) samples showed to be reactive for influenza viruses: 36 of type A and one of type B; and from remaining 568 samples, 254 tested negative, 122 were inconclusive, and 192 were positive for other respiratory viruses (HRV, RSV, hMPV, PIV and Adv), which were the subject of other studies [9,12-15]. Among the negative and inconclusive samples, 218 were tested by RT-PCR for the amplification of HA and NA gene segments and three other positive samples for influenza virus type A were detected. The remaining 158 samples were not tested due to insufficient amount. Therefore, the total prevalence found for these viruses in the study was 6.6% (40/605).

Most children were outpatients (33/40; 82.5%), males (26/40; 65%; p = 0.013), and the median age was 14.5 months, ranging from one to 60 months. Influenza cases peaked in July, though they were observed from February to September in the time period of the study.

Out of the 40 positive samples, the HA sequence amplification by subtype-specific PCR was obtained from 25 of them: 16 were subtyped as H3, eight as H1, and one was characterized as type B. Regarding NA sequence, four specimens were subtyped as N1 and 24 as N2. The subtyping of influenza A viruses revealed that 41.0% (16/39), 17.9% (7/39) and 2.6% (1/39) of infections were attributed to H3N2, H1N2 and to H1N1 viruses, respectively [see Additional file 1].

Influenza viruses H1N2, H3N2, and B were found simultaneously in Uberlândia during the 2002 flu season. Also during that year, though HA sequence from four samples was not obtained, three viruses were identified as subtype N1 and one as N2. Viruses with N1 subtype also circulated in 2006 and 2009.

### Comparative analysis of HA and NA sequences

For further characterization of the influenza viruses found in Uberlândia, PCR products of HA sequence from 25 samples and of NA sequence from 24 samples were purified and sequenced. By using the BLAST tool, it was observed that all Uberlandia’s strain sequences showed high identity with sequences of influenza viruses that had circulated previously in other regions of the world [see Additional file 1]. Among eight HA sequences of influenza virus subtype H1, obtained from samples collected in 2002, six (213, 214, 215, 228, 232, and 241) were identical to each other in the deduced amino acid sequence and showed highest identity to an isolate that circulated in New York in the same year.

**Figure 1:**
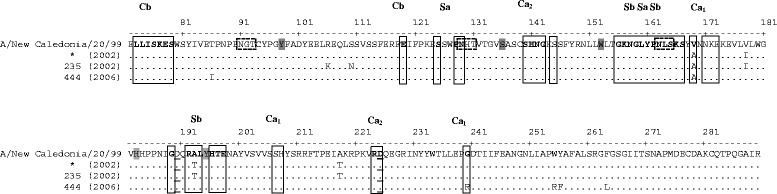
**Alignment of the deduced amino acid sequences of domain HA1 of subtype H1 of viral isolates found in Uberlândia.** HA1 domain sequences of 2002 H1N2 and 2006 H1N1 viruses of samples collected in Uberlândia were compared to the sequence of the vaccine strain A/New Caledonia/20/99 (accession number AJ344014) that was available in the same period of sample collection. Residues that match the residues of the vaccine sequence shown at the top are represented by dots and those divergent are shown. Residues involved in receptor binding are shaded, sialic acid binding sites are underlined, antigenic sites Ca_1_, Ca_2_, Cb, Sa and Sb are shown as open boxes, and *N*-linked glycosylation sites are marked as dashed open boxes. The numbering above the alignment represents the positions of amino acid residues of H1 protein according to the reference sequence A/New York/399/2003 (H1N1) (accession number CY002808). * sequences of viruses detected in samples 213, 214, 215, 228, 232, and 241.

Analysis of the HA1 domain sequence of H1 viruses found in this study in comparison with the sequence of the strain A/New Caledonia/20/99, which composed the flu vaccine available from 2000 to 2007, showed that those found in 2002 had higher nucleotide and amino acid identities (97.9-98.5 and 97.2-98.2%, respectively) than the one collected in 2006 (97.3 and 97.2%, respectively). Analysis of important antigenic sites in this domain (sites Ca, Cb, Sa and Sb) revealed a few substitutions in the deduced amino acid sequence compared to the vaccine strain sequence (Figure 1). All H1 viruses of this study showed an Val to Ala substitution at position 169, which is located within the catalytic site 1 (Ca_1_), and the virus collected in 2006 (sample 444) had also the substitution Gly to Arg at position 240 in the same site. Moreover, the viruses collected in 2002 had the substitution Ala to Thr at position 193 in the Sb site.

**Figure 2:**
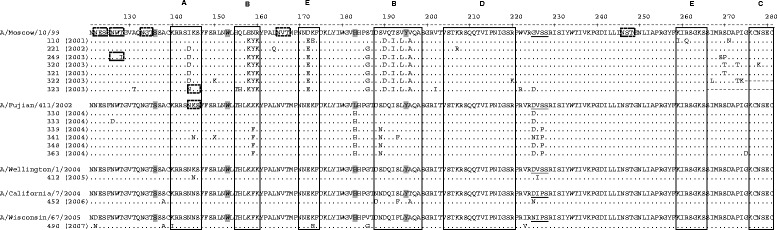
**Alignment of the deduced amino acid sequences of domain HA1 of subtype H3 of viral isolates found in Uberlândia.** Sequences of H3N2 viruses of samples collected from 2001 to 2007 were compared to the strains of the vaccine available in each corresponding year: A/Moscow/10/99 (accession number DQ487341; 2001–2003 vaccine), A/Fujian/411/2002 (accession number CY112933; 2004 vaccine), A/Wellington/1/2004 (accession number CY012104; 2005 vaccine), A/California/7/2004 (accession number CY114373; 2006 vaccine), and A/Wisconsin/67/2005 (accession number CY114381; 2007 vaccine). Residues that match the residues of the vaccine strain sequence, which is shown at the top of each group of sequences separated by time period, are represented by dots and those that diverge are shown, *N*-linked glycosylation sites are marked by dashed boxes (including loss of a glycosylation site), residues involved in receptor binding are shaded, sialic acid binding site is underlined and the antigenic sites A-E are shown as open boxes. The numbering above the alignment represents the positions of amino acid residues of H3 protein according to the reference sequence A/Taiwan/30005/2004(H3N2) (accession number DQ249261).

Similarly, analyses performed with HA domain 1 sequences of influenza viruses of subtype H3 and revealed that only three (348, 452 and 490) out of 16 nucleotide sequences were identical to sequences of isolates that circulated in South America in the same years of the samples collected in this study [see Additional file 1]. Three other samples (323, 333 and 412) had viruses with their deduced amino acid sequences identical to isolates found in the same year or in the immediately preceding year in other countries. In comparison with sequence of A/Moscow/10/99 H3N2 strain, which composed the southern hemisphere (SH) vaccine distributed in the 2001–2003 period, the identities in nucleotide and amino acid sequences varied from 94.5% and 89.0%, respectively (sample 323), to 96.7% and 93.2%, respectively (sample 321), both collected in 2003 (Figure 2).

Amino acid changes were observed in all antigenic sites (A through E) in the viruses collected in the 2001–2003 period and those in antigenic sites B and E were found in all of these viruses. Moreover, the majority of the H3 viruses contained substitutions in three antigenic sites. Viruses from samples 110 and 323 showed the highest number of substitutions in the antigenic sites (11 substitutions), while those from samples 221, 320, and 322 showed changes in the highest number of sites (four out of five sites).

Substitutions at position 105 in sample 221 (data not shown) and position 144 in sample 323 generated additional *N*-linked glycosylation sites in the hemagglutinin sequences (Figure 2). In the latter, the extra glycosylation site was located within the antigenic site A. A loss of a glycosylation site was observed at position 126 in sample 249.

Nucleotide and amino acid sequences of the viruses detected from 2004 to 2007, in comparison to the vaccine strain available in the respective years of the sample collection, showed identities that varied from 96.7 to 99.1% and from 95.3 to 98.9%, respectively (Figure 2).

Regarding antigenic sites, in comparison with the sequence of vaccine strain available in the corresponding year of sample collection, the majority of substitutions of viruses detected in the six samples collected in 2004 concentrated within site B (Figure 2). Also, a substitution in the receptor binding site was observed (Leu to His at position 183) and the virus of sample 341 contained one substitution in site A; the sequence of sample 412, found in 2005, contained one substitution at site A and another (Val to Ile) in the binding site of sialic acid; the sequence of sample 452 of 2006 showed three substitutions in the antigenic site B; the H3 sequence of specimen 490 of 2007 presented one substitution located in site A and another in site E. The amino acid residue at position 226, in the binding site of terminal sialic acid, was Ile, a substitution that was also observed with other isolates (samples 341/2004, 412/2005, and 452/2006).

In regard to neuraminidase, the nucleotide sequence of sample 444, characterized as N1, had the highest identity with a strain (A/Tennessee/UR06-0236/2007) that circulated in the northern hemisphere in the same year of the sample collection [see Additional file 1]. The comparison with the sequence of the vaccine strain A/New Caledonia/20/99, recommended for 2000–2007 period, showed high nucleotide and deduced amino acid identities (98.3 and 98.0%, respectively), and the divergent amino acid residues were at positions 187 (M, in the vaccine strain, and K in the isolate found in this study); 286(T-K); 331(E-K); 374(W-R) and 377(N-Y).

N2 nucleotide sequences from 22 H1N2 and H3N2 samples were obtained and analyzed by BLAST. Twelve of them (213, 214, 215, 228, 232, 235, 322, 323, 330, 333, 358, and 490) had either nucleotide or deduced amino acid sequences identical to isolates found somewhere else in the same year or in the preceding year, and the sequence of sample 341 was identical to the sequence of a strain that circulated two years later in the southern hemisphere [see Additional file 1]. The nucleotide and deduced amino acid identities, in comparison with the vaccine strains released from 2000–2007, varied from 97.7 and 96.9% (samples 320/321 obtained in 2003), respectively, and 98.9% and 99.4% (sample 452 of 2006), respectively. The conflicting amino acid residues are shown in Table 2.

**Table 2:** **Amino acid divergent positions in neuraminidase sequences of N2 influenza isolates found in Uberlândia between 2001 and 2007 compared with sequences of the vaccine strains of 2001–2003, 2004, 2005, 2006 and 2007 seasons**

**Virus strain/year**	**Amino acid variation sites**																			
	**133**	**141**	**150**	**160**	**167**	**172**	**194**	**199**	**216**	**221**	**250**	**254**	**265**	**267**	**271**	**285**	**298**	**307**	**310**	**332**
**A/Moscow/10/99** ^**a**^		N		M	P	K	V	E	G	K	A	I	T	P	S	P	G	V	Y	
110/2001		D			L															
* /2002		N						K						L						
221/2002		N				R		K					I	T						
241/2002		N		K				K						L			C			
249.322/2003									G	K			I	T	S			V		
320.321/2003									G	N			I	T	N			V		
323/2003									V	K			T	T	S			I		
**A/Fujian/411/2002** ^**b**^											A		T					I		S
330.333/2004											A		I					V		F
341.348/2004											A		T					I		S
339/2004											T		T					I		S
**A/Wellington/1/2004** ^**c**^	A									K		I							Y	
412/2005	G									E		V							W	
**A/California/7/2004** ^**d**^						K														
452/2006						R														
**A/Wisconsin/67/2005** ^**e**^			H				V									L			Y	
490/2007			R				I									P			H	

*Samples 213, 214, 215, 228, 232, 235; ^a^DQ487331; ^b^CY112935; ^c^EF512573; ^d^CY114375; ^e^CY114383.

Regarding the catalytic and framework sites within the NA sequence, the analysis showed that they were conserved in all H1N1 and H3N2 isolates of this study (composition of the catalytic site is R118, D151, R152, R224, E276, R292, R371, and Y406 and composition of the framework sites is E119, R156, W178, S179, D/N198, I222, E227, H274, E277, N294 and E425). Nevertheless, amino acid variations were observed in other positions (data not shown). None of the isolates carried a known oseltamivir resistance mutation in the NA sequence (positions 151, 152, 222, 224, 274, 276, 292 and 294).

## Discussion

Until the present study, there was no information regarding the strains of influenza viruses circulating in Uberlândia. The circulation of influenza viruses was investigated during a period of ten years and the isolates were characterized and compared with other strains.

By using immunofluorescence assay, 6.1% specimens was tested positive for influenza viruses. A similar rate (6.3%) was observed in a study in northeastern Brazil [16] by using the same procedure. Still in our country, studies carried out with children’s clinical samples, by testing samples with IFA, cell culture isolation and/or RT-PCR, lower rates of infection caused by influenza viruses were reported, ranging from 1.2% - 5.0% [17-20]. On the other hand, in adults, higher rates (17.6% - 40.0%) of respiratory disease caused by this virus have been reported [21,22]. Still, despite lower frequencies, it is important to emphasize the role of children in the transmission of influenza viruses in the community, since they shed the pathogen longer and in higher amounts than adults [23].

Even though PCR may have higher sensitivity than IFA [24-26], in this study not all samples that were tested positive for influenza viruses by IFA were positive by RT-PCR. One of the main reasons for the lower sensitivity of RT-PCR was probably due to RNA degradation from long-term storage [27], especially those stored at −70°C for approximately ten years.

In this study, we detected infections caused by influenza viruses from the end of summer to the end of winter, and this observation was consistent with the findings reported by Carraro et al. [28] during 2001–2003 influenza seasons in São Paulo state. Besides, the number of influenza cases observed in the 2001–2003 period in Uberlândia was higher than in other flu seasons, which may be the cause of the higher rate of influenza-related morbidity reported in other parts of the world [29].

H1N1 and H3N2 are usually the most common influenza A subtypes found infecting humans [30], and H3N2 viruses have been the dominant strain in most years since they first emerged in 1968. Still, this subtype had caused one of the most serious respiratory infections [31-33] until 2009, when the novel swine-origin influenza H1N1 virus emerged and was responsible for the new pandemic [34]. In this study, H3N2 also predominated, with 41.0% of isolates that were subtyped, and was responsible for the majority of hospitalizations (in five out of six hospitalizations).

The co-circulation of influenza virus subtypes is found commonly and in the 2002 season in Uberlândia, H3N2 co-circulated with subtype H1N2 and influenza B virus, which was detected in only one clinical specimen. Chieochansin et al. [35] also reported only one case involving influenza B virus in 302 samples collected from hospitalized pediatric patients. A fourth viral subtype, the H1N1, may have also circulated in 2002, since NA sequences of three clinical samples were characterized as N1. However, RNA of HA in these samples was not detected,

In the 2000–2002 period, several H1N2 reassortant viruses were isolated in different parts of the world [36-38] and were also found in our study. In Brazil, in the north region, H1N2 cases were detected between January and April 2003 (epidemiological weeks 2 to 14) [Personal Communications, Wyller Mello and Mirleide Santos], whereas in Uberlândia, which is located in the southeastern Brazil, these strains circulated as early as July 2002.

Sequence analyses of subtypes H1N1, H1N2 and H3N2 and influenza B virus found in this study showed high identities with variants found in other continents in previous years, such as in Asia, Africa and North America, suggesting a global origin of new strains, which are then spread to other countries through human mobility [39,40].

Every year, WHO recommends specific vaccine strains for influenza vaccine production. This strategy needs to be followed since hemagglutinin and neuraminidase are under selective pressure and undergo frequent antigenic changes in order to evade the host’s immune system [4]. Comparison of the deduced amino acid sequences of Uberlandia’s isolates and those of the vaccine strains showed that they were related closely in the majority. Precisely at antigenic site A of domain HA1, the position 144 showed higher variability, with Ile in the virus detected in 2001, changing to Asp in 2002/2003, and to Asn, in 2003/2004, corroborating with Bragstad et al. [41], who reported that after 2002, this was one of the regions in which the highest number of substitutions in H3 subtype virus was detected. Also, divergences from nine to 11 amino acid residues in three or four antigenic sites, by comparing the HA sequence of the vaccine strain and those of the viruses detected between 2001–2003, may have caused a certain decrease in protection efficiency of the vaccine that was available in the same time period in Brazil – possibly configuring a mechanism of immune evasion by these variants [42].

For H1 subtype, important amino acid changes in the HA sequence are clustered in the variable antigenic sites, which have been proposed based on sequence homology to the H3 sites B + D, B, C + D + A and E, respectively [43]. In the present study, we detected small numbers of variations in the Ca and Sb sites in the HA of viruses detected in 2002 and 2006 in comparison to the vaccine strain. In H1 isolates, we found Gln and Gly at positions 226 and 228, respectively. These determinants, however, have been reported in H1 viruses from both human and avian hosts [44]. In addition, H1 D190 and D225 determinants of receptor-binding specificity of H1N1 from humans [45] were also observed.

The antigenic regions A through E of the HA1 domain are potential targets for neutralizing antibodies, and amino acid changes in these regions have been associated with annual epidemics in humans. Still, it has been proposed that epidemiologically important drift variants generally exhibit four or more amino acid substitutions located in two or more antigenic sites in this domain [46]. These substitutions may generate a new strain against which immunity acquired previously, by either infection or vaccination, is no longer effective [47]. On the other hand, the receptor binding sites are likely to resist changes [48]. Our results showed that all of the 2001–2003 H3 sequences presented at least eight substitutions located at no less than two antigenic sites. In regard to HA protein binding to sialic acid, Leu and Ser at positions 226 and 228, respectively, have been reported to be important in H3 subtypes and are determinants of influenza viruses that infect humans [44]. In this study, at position 226, we found Val in the H3N2 viruses found in the 2001–2004 period and Ile in the 2004–2007 period (Figure 2). Nevertheless, Val and Ile, like Leu, are neutral, non-polar amino acids and thus the presence of any of them may have preserved the properties of the sialic acid binding pocket of HA, as suggested by others [49]. Still, mutations at this position may represent a selective advantage to the virus [50].

Increasing number of *N*-linked glycosylation sites have been shown to attenuate H3N2 influenza viruses in a mouse model [51]. On the other hand, addition or removal of a glycosylation site may generate virus variants that evade the host immune system, including the response generated by vaccination, and maintain a sustained circulation of the virus within the human population [52]. We found one isolate (323, Figure 2) in a sample collected in 2003 that contained an additional glycosylation site located in an antigenic site, when compared to the vaccine strain.

Concerning the NA, variations that promote modifications in the antigenic properties usually occur in the catalytic or framework sites of the protein [53]. The amino acid substitutions found in the NA sequences of the Uberlândia isolates, in comparison with the sequences of the vaccine strains of each corresponding year, were not located in these sites and, therefore, may not have led to significant antigenic changes.

Circulation of naturally resistant H1N1 viruses to oseltamivir has been reported elsewhere, even in the absence of selective drug pressure [54]. The extensive use of this antiviral, especially after the emergence of the pandemic H1N1 virus, in 2009, may select and increase the frequency of resistant variants in flu cases. In this study, oseltamivir-resistant viruses were not detected. It should be noted, however, that the sequencing was performed with RT-PCR products, using RNA extracted from nasopharyngeal secretions. This test does not detect the presence of low-frequency viral variants that are resistant to this antiviral in a sample.

## Conclusions

These findings constitute the first molecular epidemiological study of influenza viruses in Uberlândia city, southeastern Brazil, and are going to be used as a reference for future studies. The genetic changes found in this investigation, mainly those characterized in important antigenic sites, justify the continued efforts to monitor the emergence of new viral variants in an attempt to reduce the burden that they may cause to public health.

## Additional file

Additional file 1: Table S1. Demographic data and isolates with high identity in HA and NA sequences.

## Abbreviations

ARD: Acute respiratory disease
NPA: Nasopharyngeal aspirates
IFA: Indirect immunofluorescence assay
RSV: Respiratory syncytial virus
HRV: Human rhinovirus
hMPV: Human metapneumovirus
PIV: Parainfluenza virus
Adv: Adenovirus
RT-PCR: Reverse transcription-polymerase chain reaction
HA: Hemagglutinin
NA: Neuraminidase
BLAST: Basic local alignment search tool
SH: Southern hemisphere
WHO: World Health Organization
