# Supplementary material for: Molecular characterization of influenza viruses collected from young children in Uberlandia, Brazil - from 2001 to 2010
Source: BMC Infect Dis. 2015 Feb 18;15:71. doi: 10.1186/s12879-015-0817-z (PMC4336712; doi:10.1186/s12879-015-0817-z)
Supplement: Additional file 1: Table S1. — Demographic data and isolates with high identity in HA and NA sequences. [file 12879_2015_817_MOESM1_ESM.docx]

**Additional file 1**

**Table S1 - Demographic data and isolates with high identity in HA and NA sequences.**

| **Sample number** | **Date of collection**  **(mm-aa)** | **Place of sample collection** | **Type / subtype** | **HA sequence isolates**  **(% identity: nucleotides; amino acids)** | **NA sequence isolates**  **(% identity: nucleotides; amino acids)** |
| --- | --- | --- | --- | --- | --- |
| 35 | 03/2001 | PFA | A ND |  |  |
| 76 | 05/2001 | PFA | A ND |  |  |
| 85 | 05/2001 | PFA | A ND |  |  |
| 86 | 05/2001 | PFA | A ND |  |  |
| 110 | 06/2001 | PW | A H3N2 | A/RiodeJaneiro/465/01 (H3N2)  (99.1; 97.9) | A/Missouri/NHRC0001/2000(H3N2)  (99.5; 99.5) |
| 180 | 05/2002 | PFA | A N2 |  |  |
| 185 | 05/2002 | PFA | A N1 |  |  |
| 188 | 05/2002 | PFA | A ND |  |  |
| 191 | 05/2002 | EDP | A ND |  |  |
| 193 | 05/2002 | PW | A N1 |  |  |
| 194 | 05/2002 | EDP | A N1 |  |  |
| 212 | 06/2002 | PFA | A ND |  |  |
| 213 | 07/2002 | PFA | A H1N2 | A/New York/78/2002 (H1N2)  (99.2;100.0) | A/Hawaii/10/2002(H1N2)  (100.0; 100.0) |
| 214 | 07/2002 | PFA | A H1N2 | A/New York/78/2002 (H1N2)  (99.8; 100.0) | A/Hawaii/10/2002(H1N2)  (100.0; 100.0) |
| 215 | 07/2002 | PFA | A H1N2 | A/New York/78/2002 (H1N2)  (99.7; 100.0) | A/Hawaii/10/2002(H1N2)  (100.0; 100.0) |
| 221 | 07/2002 | PFA | A H3N2 | A/Denmark/6/2002 (H3N2)  (99.5; 98.9) | A/Netherlands/120/2002(H3N2)  (99.8; 99.4) |
| 223 | 07/2002 | PFA | A ND |  |  |
| 228 | 07/2002 | PFA | A H1N2 | A/1660/02/2002 (H1N2)  (99.7; 100.0) | A/Hawaii/10/2002(H1N2)  (100.0; 100.0) |
| 232 | 08/2002 | PFA | A H1N2 | A/New York/78/2002 (H1N2)  (99.7; 100.0) | A/Hawaii/10/2002(H1N2)  (99.8; 100.0) |
| 235 | 08/2002 | PFA | A H1N2 | A/New York/78/2002 (H1N2)  (99.2; 99.1) | A/Hawaii/10/2002(H1N2)  (100.0; 100.0) |
| 237 | 08/2002 | PFA | B | B/RiodeJaneiro/209/02  (99.8; 100.0) |  |
| 241 | 09/2002 | PFA | A H1N2 | A/New York/78/2002 (H1N2)  (99.7;100.0) | A/Hawaii/10/2002(H1N2)  (99.4; 98.8) |
| 249 | 02/2003 | PFA | A H3N2 | A/EspiritoSanto/88/02 (H3N2)  (99.3; 98.4) | A/New York/409/2002(H3N2)  (99.7; 99.2) |
| 320 | 07/2003 | PFA | A H3N2 | A/Brazil/BR-IAL-2302/2005 (H3N2)  (98.8; 98.2) | A/Denmark/4/2002(H3N2)  (99.7; 99.2) |
| 321 | 07/2003 | PFA | A H3N2 | A/Texas/NHRC0001/2002 (H3N2)  (99.2; 98.3) | A/Denmark/4/2002(H3N2)  (99.7; 99.2) |
| 322 | 07/2003 | PFA | A H3N2 | A/Dunedin/12/2002 (H3N2)  (99.1; 98.4) | A/New York/409/2002(H3N2)  (100.0;100.0) |
| 323 | 07/2003 | PW | A H3N2 | A/Bangladesh/C5-16/2003 (H3N2)  (99.6; 100.0) | A/Netherlands/213/2003(H3N2)  (100.0; 100.0) |
| 330 | 03/2004 | PFA | A H3N2 | A/Brazil/BR-IAL-2302/2005 (H3N2)  (99.6; 99.5) | A/Finland/303/2003 (H3N2)  (99.6; 100.0) |
| 333 | 03/2004 | PICU | A H3N2 | A/Paraguay/PY-697/2004 (H3N2)  (99.8; 100.0) | A/Scotland/81/2003(H3N2)  (100.0; 100.0) |
| 339 | 03/2004 | PFA | A H3N2 | A/RiodeJaneiro/17/04 (H3N2)  (99.5; 99.5) | A/England/558/2003(H3N2)  (99.6; 98.9) |
| 341 | 03/2004 | PFA | A H3N2 | A/Brazil/BR-1898-ORIGINAL/2006 (H3N2)  (99.7; 99.5) | A/SANTIAGO/9491/2006(H3N2)  (100.0; 100.0) |
| 348 | 04/2004 | PFA | A H3N2 | A/StaCatarina/380/04 (H3N2)  (100.0; 100.0) | A/England/558/2003(H3N2)  (99.6; 100.0) |
| 363 | 05/2004 | NICU | A H3N2 | A/Argentina/AG-R126-04/2004 (H3N2)  (99.3; 99.0) | - |
| 412 | 07/2005 | PFA | A H3N2 | A/Texas/TX-VC4-775/2004 (H3N2)  (99.8; 100.0) | A/Niigata/494/2005(H3N2)  (99.3; 98.7) |
| 435 | 05/2006 | PFA | A N1 |  |  |
| 442 | 06/2006 | PFA | A N1 |  |  |
| 444 | 06/2006 | PICU | A H1N1 | A/Juliaca/FLU3973/2006 (H1)  (99.8; 99.5) | A/Tennessee/UR06-0236/2007(H1N1)  (99.1; 98.8) |
| 452 | 07/2006 | PFA | A H3N2 | A/Brazil/BR-1898-ORIGINAL/2006(H3N2)  (100.0; 100.0) | A/California/NHRC0007/2005(H3N2)  (99.6; 99.4) |
| 490 | 07/2007 | PICU | A H3N2 | A/Maracay/FLU7827/2007 (H3N2)  (100.0; 100.0) | A/Brazil/80/2007(H3N2)  (100.0; 100.0) |
| 531 | 04/2009 | PFA | A N1 |  |  |
